# Pre-operative simulation using a three-dimensional printing model for surgical treatment of old and complex tibial plateau fractures

**DOI:** 10.1038/s41598-020-63219-w

**Published:** 2020-04-08

**Authors:** Sheng Shen, PeiZhao Wang, XiaoYong Li, Xu Han, HongLue Tan

**Affiliations:** Department of Knee Surgery, Henan LuoYang Orthopedic-Traumatological Hospital(Henan Orthopedic Hospital), Qiming Southern Road, Luoyang, Henan 471002 P.R. China

**Keywords:** Trauma, Fracture repair, Outcomes research

## Abstract

To investigate the clinical efficacy of pre-operative simulation using a three-dimensional (3D) printing model for surgical treatment of old and complex tibial plateau fractures. Forty-two patients with old and complex tibial plateau fractures were retrospectively reviewed from January 2014 to January 2018, which were divided into a conventional planning group (n = 22) and a planning with 3D printing group (n = 20). In the planning with 3D printing group, preoperative equal-ratio fracture models prepared using the 3D printing technique were used to perform pre-operative simulation and guide the real surgical operation. In the conventional planning group, the operation was performed based on pre-operative computed tomography (CT) images. Surgery duration, blood loss and the number of fluoroscopy during operations were recorded. During follow-up, the quality of fracture reduction and complications were also recorded. Knee functions were evaluated using the hospital for special surgery (HSS) scoring system. The operation time, blood loss and the number of fluoroscopy during operation in the planning with 3D printing group were less than that in the conventional planning group (*P* < 0.01). All patients were followed up for mean of 24.38 ± 7.62 months. The rate of excellent fracture reduction in the planning with 3D printing group and conventional planning group was 75% and 45.45%, respectively (*P* = 0.05). The complication rate was 15% in the planning with 3D printing group and 31.82% in the conventional planning group. At the final follow-up evaluation, the mean HSS score was 86.05 ± 7.67 in the planning with 3D printing group and 79.09 ± 6.75 in the conventional planning group (*P* = 0.003). The rate of excellent results in the planning with 3D printing group was 70% and in the conventional planning group was 45.45% (*P* = 0.083). In conclusion, pre-operative simulation using a 3D printing model may be helpful for the treatment of old and complex tibial plateau fractures, which may be conducive to the pre-operative planning and to making the surgical procedure accurate and personalized. However, its clinical effectiveness need to be further assessed by a prospective randomized-controlled study.

## Introduction

Complex tibial plateau fractures are mostly caused by high-energy injuries and are often accompanied with the collapse of the tibial plateau surface, displacement of the comminuted fragments and knee joint dislocation. If this type of fracture is not treated properly, complications such as postoperative wound infection, skin necrosis, joint deformity and traumatic arthritis are very likely to occur^1^. If complex tibial plateau fractures develop into old fractures, they become more challenging for orthopedic surgeons to optimize the surgical procedures to improve the operation efficiency and to reduce postoperative complications^2^. In general, for complex tibial plateau fractures, the surgical planning is mainly based on pre-operative X-ray and CT images. Although three-dimensional (3D)-CT can display the spatial position relationship of the fracture fragments, the pre-operative design is still limited because of the 2D-display of each image, which makes it difficult to achieve satisfactory anatomical reduction of fracture fragments during operation^3^. For old fractures, it is more difficult to identify and reposition the comminuted fragments according to conventional pre-operative planning, which inevitably leads to prolonged operation time, increased blood loss and postoperative complications.

After reduction of old and complex tibial plateau fractures, reliable plate fixation matched with the shape of the proximal tibia is another guarantee to obtain satisfactory post-operative knee functions^4^. A good match between the plate and the anatomically reduced bone surface can undoubtedly obtain better biomechanical properties of internal fixation and more reliable long-term clinical efficacy. However, the clinical reality is that the present proximal tibial anatomical plate does not always match the bone shape due to the differences in the bone morphology of patients; it is therefore necessary to prepare enough types of plates preoperatively to meet intraoperative use. Regardless, the plates may still be “non-anatomic” during operation, resulting in the need to reshape the plate temporarily and repeatedly, which further prolongs the operation time and increases the blood loss and fluoroscopy time during operation. Therefore, according to the specific situation of tibial plateau fractures for each patient, plate fixation should be personalized to achieve the optimal fixation effect using the present plates. In recent years, pre-operative 3D printing assisted surgery based on digital medicine provides an opportunity to solve this problem^3–6^.

3D printing is a technology based on digital model, in which Mimics 3D reconstruction software and a 3D printer are used to print out required objects with bondable materials such as powdered metal or plastic through a layer-by-layer printing method^6^. 3D printing technology has been recently applied in the medical field, especially showing potential advantages in the accuracy and efficiency of assisted orthopedic surgery^5,6^. In this study, the digital model of old and complex tibial plateau fractures is converted into a real printing model with a 1:1 ratio through 3D printing technology. The specific morphological structure and displacement of the fragments are accurately and comprehensively observed through the model. Preoperative simulation operation is performed on the printed fracture model to accurately reduce the fracture fragments, correct various rotation and angulation deformity, and restore the anatomical morphology of the proximal tibial structure. Then the appropriate plates are selected and reshaped for fixation. During the operation, the reduction and fixation of fractures are strictly completed according to preoperative simulation planning, thus improving the operation efficiency and optimizing the operation effect.

Unlike previous studies that focused on fresh tibial plateau fractures^3^, in this study, we hypothesized that pre-operative simulation using a 3D printing model could reduce operation time and blood loss and significantly improve the surgical, clinical and radiographic outcomes of old and complex tibial plateau fractures. Thus, the aim of the study was to compare the outcomes between patients treated with and without the assistance of the 3D printed models and to evaluate the feasibility and effectiveness of 3D printing model simulation for preoperative planning.

## Materials and Methods

### Study design and participants

A retrospective study was designed to enroll 51 patients with old and complex tibial plateau fractures who underwent operative treatment from January 2014 to January 2018. The inclusion criteria were as following: (1) Patients ranged from 25 years to 55 years; (2) patients had old tibial plateau fractures (in this study, all patients accepted vessel B-ultrasound examination of the injured lower extremity after admission, showing formation of deep vein thrombosis in the anterior or posterior tibial vein, popliteal vein, peroneal vein and intermuscular vein of the calf. The patients accepted systemic anticoagulant and local venous thrombolytic therapy for more than 3 weeks, so these fractures all developed into old fractures); (3) patients had complex fracture of either type IV, V or VI according to the Schatzker classification^7^; (4) the preoperative planning was based on 3D-CT images or a 3D printing model; and (5) at least a 12-month follow-up. The exclusion criteria were: (1) fresh or severe open fractures; (2) combined severe soft tissue injuries that needed an external fixator; and (3) incomplete follow-up data.

**Figure 1:**
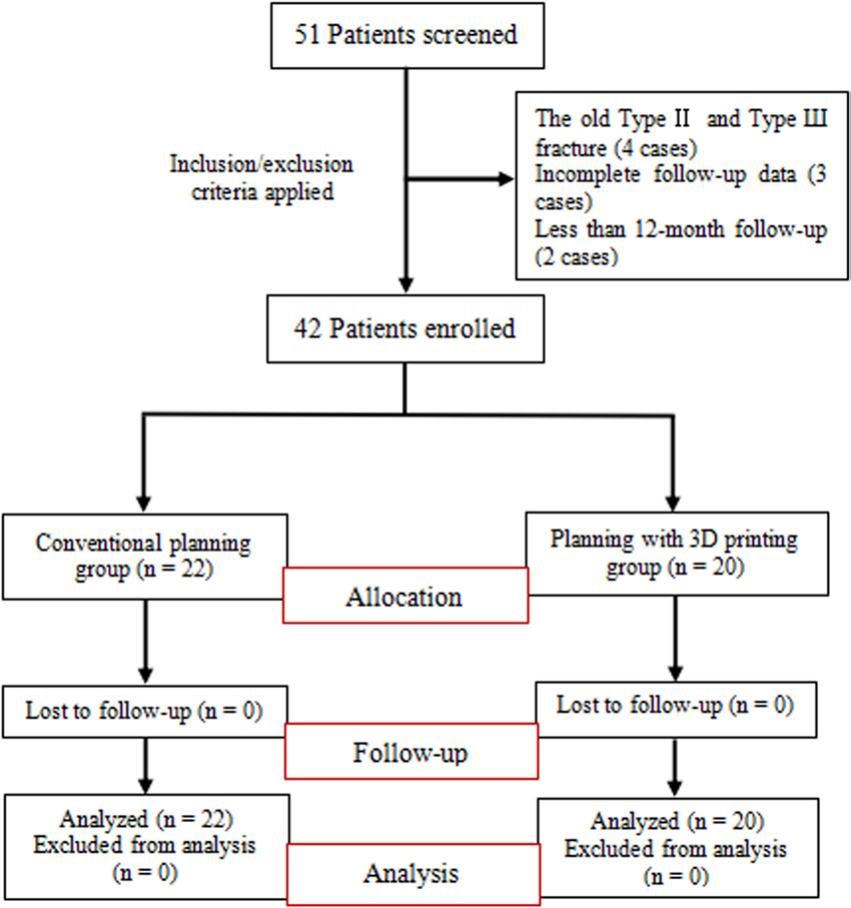
The flowchart of the study patients.

Based on aforementioned criteria, 4 cases were type II and III fracture, 3 cases had incomplete follow-up data, and 2 cases had follow-up time less than 12 month. Thus 42 patients were included in this study. There were 27 males and 15 females with a mean age of 42.93 ± 7.69 years (ranged from 29 to 55 years). Mean body mass index (BMI) was 23.98 ± 5.08 kg/m^2^ (ranged from 20.76 to 28.04 kg/m^2^). The right knee was involved in 24 cases and the left knee in 18 cases. Seventeen patients were caused by falling from height, 15 by traffic accident, and ten by other injury. The mean time from admission to hospital to surgery was 25.83 ± 5.15 days (ranged from 21 to 40 days). According to the Schatzker classification based on preoperative X-ray photographs and CT scans, 15 were type IV, 14 were type V, and 13 were type VI^7^. Patients were divided into two groups according to the method of preoperative planning: the conventional planning group (pre- and intra-operative planning based on 3D CT images from January 2014 to September 2016) and the planning with 3D printing group (pre- and intra-operative planning based on a 3D printing model from October 2016 to January 2018). The flowchart of patients is presented in Fig. 1. The demographic data of patients are shown in Table 1.

**Table 1:** The patient’s demographic data.

		Conventional planning group (n = 22)	Planning with 3D printing group (n = 20)	*P*-value
Gender, n (%)				0.461^a^
	Male	13 (59.09)	14 (70.00)	
	Female	9 (40.91)	6 (30.00)	
BMI (kg/m^2^), M ± SD		25.02 ± 1.72	24.96 ± 2.03	0.929^b^
Age (year), M ± SD		43.00 ± 7.86	42.85 ± 7.71	0.95^b^
Side of injury, n (%)				0.721^a^
	Right	12 (54.55)	12 (60.00)	
	Left	10 (45.45)	8 (40.00)	
Cause of imjury, n (%)				0.985^a^
	Falling from height	9 (40.91)	8 (40.00)	
	Traffic accident	8 (36.36)	7 (35.00)	
	Other	5 (22.73)	5 (25.00)	
Time from admission to hospital to surgery (day), M ± SD		26.91 ± 5.65	24.65 ± 4.39	0.207^c^
Schatzker classification, n (%)				0.647^a^
	IV	9 (40.91)	6 (30.00)	
	V	6 (27.27)	8 (40.00)	
	VI	7 (31.82)	6 (30.00)	
No. of post. column plate, n (%)				0.382^a^
	0	6 (27.27)	8 (40.00)	
	1	16 (72.73)	12 (60.00)	
No. of med. column plate, n (%)	1	22 (100)	20 (100)	n/a
No. of lat. column plate, n (%)				0.461^a^
	0	9 (40.91)	6 (30.00)	
	1	13 (59.09)	14 (70.00)	
Approach method, n (%)				0.382^a^
	Antero-middle	6 (27.27)	8 (40.00)	
	Antero-middle + posteromedial	16 (72.73)	12 (60.00)	
Follow-up time (month), M ± SD		23.45 ± 8.28	25.40 ± 6.87	0.308^c^

^a^chi-squared test, ^b^Independent t test, ^c^Mann-Whitney test. M ± SD: mean ± standard deviation; n/a: not applicable; post.: posterior; med.: medial; lat.: lateral; No.: number; n: patient number.

This study was approved by the Ethics Review Committee of Luoyang Orthopedic-Traumatological Hospital (Henan Orthopedic Hospital). Informed consent was obtained from all included subjects. And informed consent was also obtained from a study participant to publish the clinical images in an online open-access publication. In addition to this, all methods were performed in accordance with the Strengthening the reporting of observational studies in epidemiology Statement and regulation of the institutional review board. Patient records/information were anonymized and de-identified prior to analysis.

### Printing model and pre-operative *in vitro* simulation

**Figure 2:**
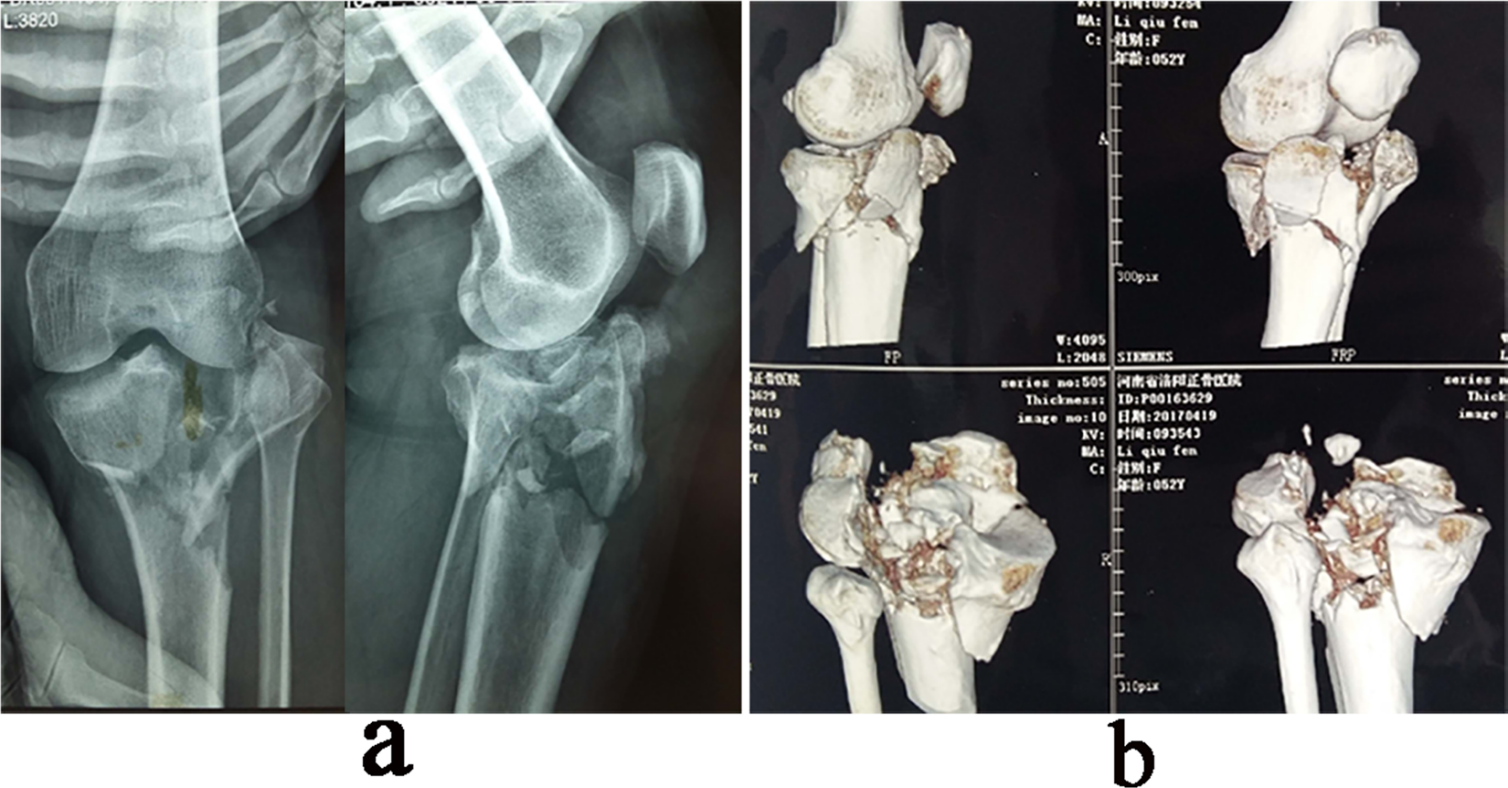
A 45-year-old female patient with an old and complex tibial plateau fracture. (**a**) Preoperative X-ray film showing the left tibial comminuted fracture (Schatzker type V). (**b)** Three-dimensional CT images of the fracture showing a complex tibial plateau fracture.

**Figure 3:**
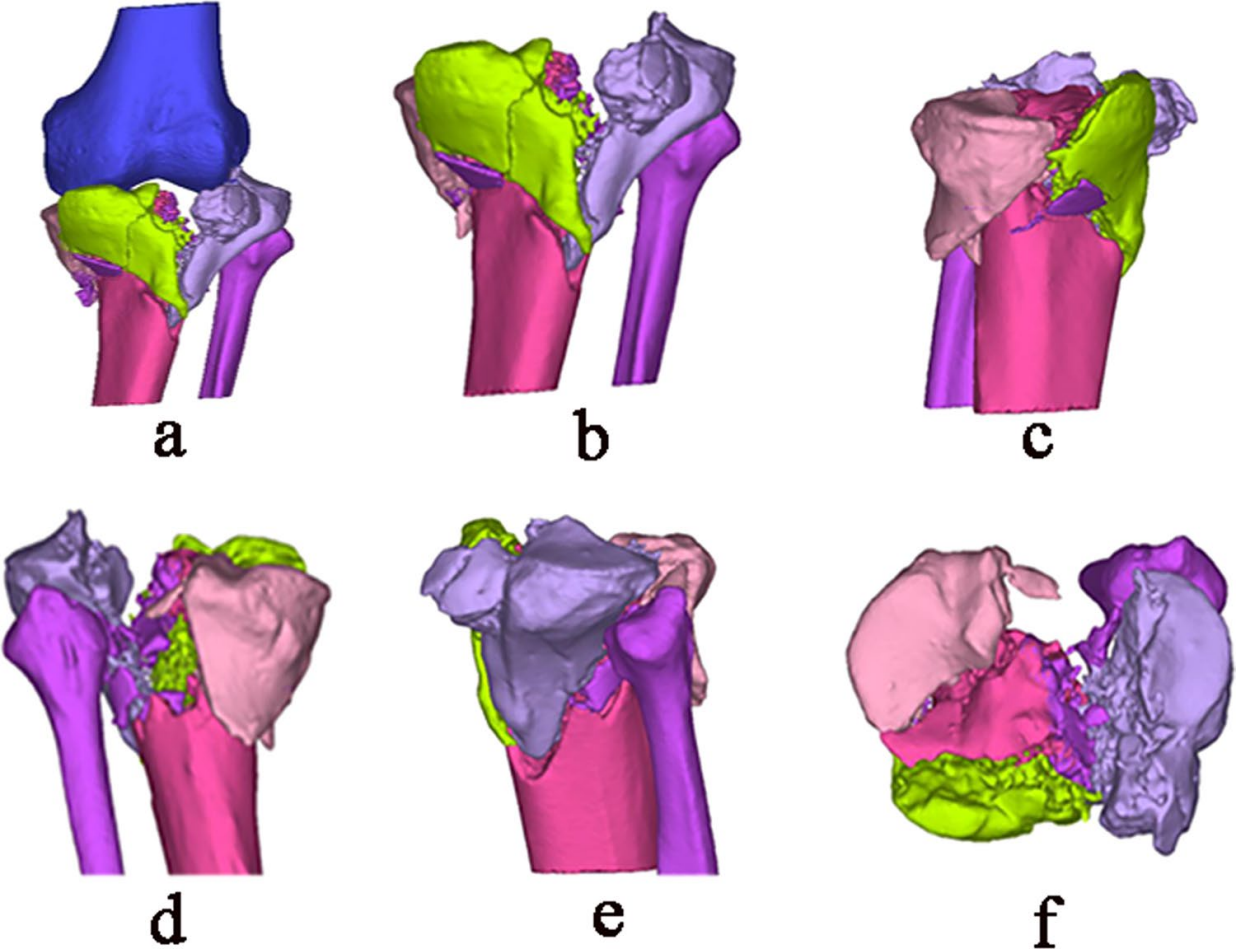
Three-dimensional reconstruction of the old and complex tibial plateau fracture in Mimics software v10.01. (**a**) Whole view of the knee joint (blue color, femoral condyle; other colors represent different fragment of the tibial plateau fracture). (**b**) Anterior view of tibial plateau fracture. (**c**) Medial view of tibial plateau fracture. (**d)** Posterior view of tibial plateau fracture. (**e)** Lateral view of tibial plateau fracture. **(f)** Cross-sectional view of tibial plateau fracture.

**Figure 4:**
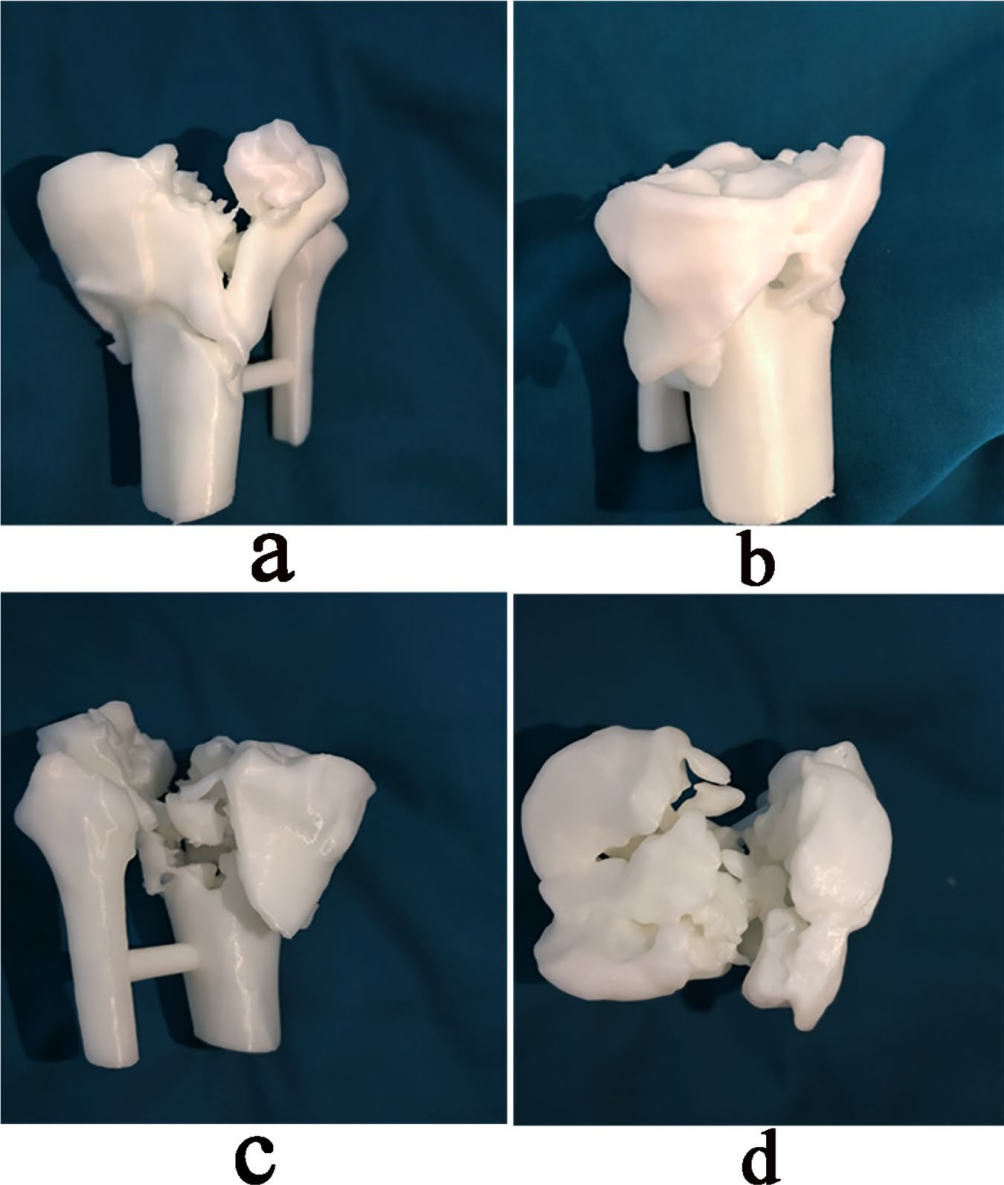
3D-printing model with a 1:1 ratio of the original tibial plateau fracture for pre-operative evaluation. (**a**) Anterior view. (**b)** Medial view. **(c)** Posterior view. (**d)** Cross-sectional view.

**Figure 5:**
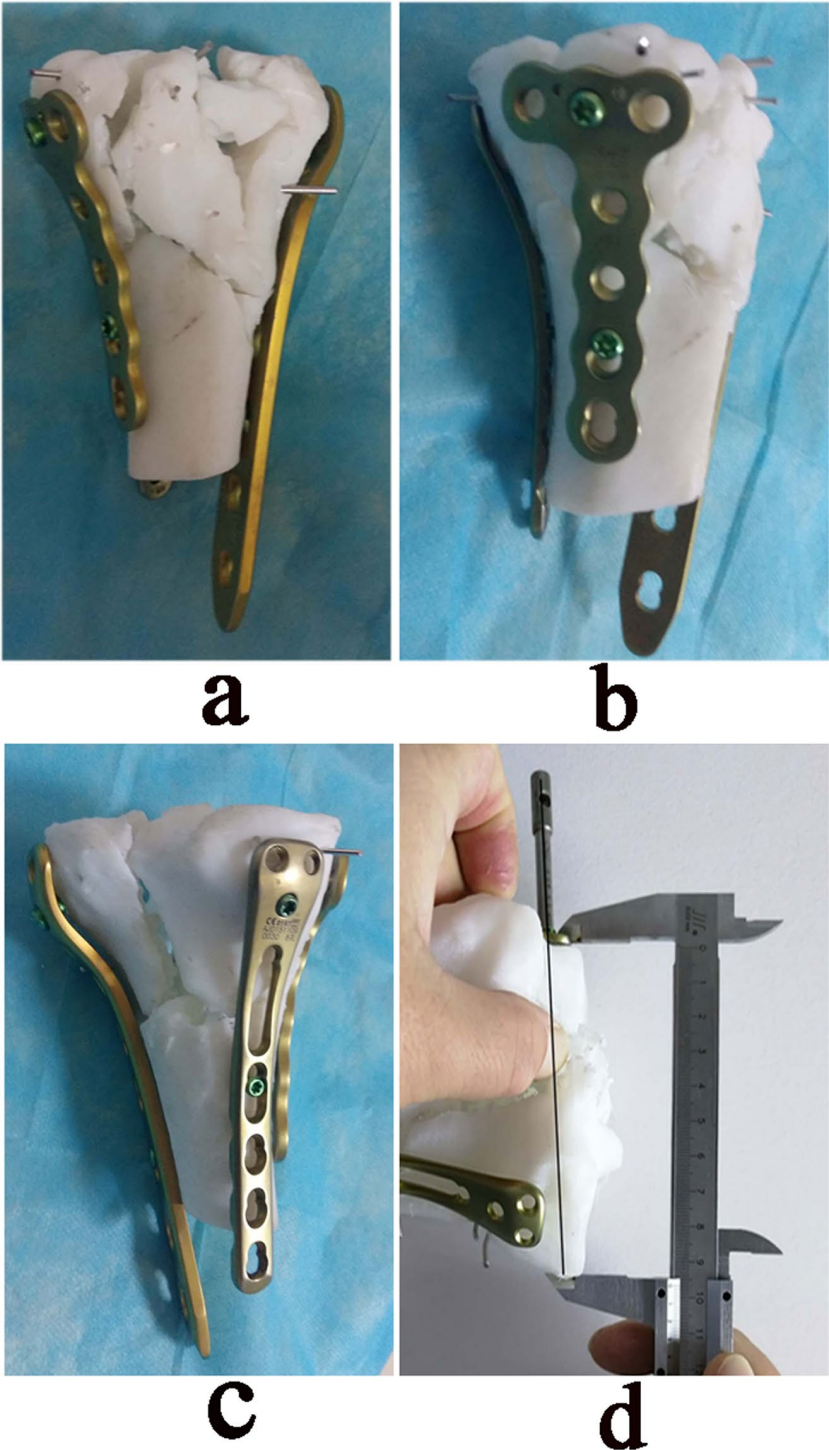
*In vitro* simulated reduction and plate fixation were performed on the printing model. (**a**) Anterior view of the model showing the lateral and medial plate fixation. (**b**) Medial view showing medial and posterior plate fixation. (**c**) Posterior view showing posteromedial and lateral plate fixation. (**d**) The plates and screw length were determined and recorded.

(1) Printing the 3D model: The tibia and fibula were scanned from the distal part of the fracture to the tibial plateau surface using a 64-slice CT with a thickness of 0.5 mm (Fig. 2). The CT data were stored in DICOM format and imported into the imaging processing software (MIMICS, version10.01, Materialise, Leuven, Belgium) to construct the 3D digital model of the fracture (Fig. 3). The model was then exported in an stereolithography (STL) format and imported to a 3D printer (3D ORTHO Waston Med Inc. Changzhou, Jiangsu, China) to produce a patient-specific fracture model with polylactic acid material (1:1 ratio of the actual fracture) (Fig. 4). (2) Operation simulation: The structural characteristics of the fracture on the 3D-printed models were clear, which was helpful in the design of the surgical plan. The steps for the reduction and fixation of the fragments were simulated on the model. First, in the printing model, the shape and displacement of each fragment was carefully observed, and the key points for fragment reduction were marked to guide the identification during simulation. Second, drilled holes along the fracture line with k-wire, and then splited the fragments with a thin bone chisel; for small fragments that had little effect on the shape of the whole reduced fracture, they could be temporarily neglected. Third, according to the anatomic mark points and lines between the fracture fragments, the fragments were reduced and fixed with k-wires temporarily. Finally, according to the fracture type, locking plates and screws were used for the fixation; the medial and posterior plates were always reshaped to match the morphology of the reduced fracture model. (Fig. 5a–c). (3) Measurement of the screw length: After plate fixation on the reduced fracture model, the length, type, location and orientation of the screws were recorded to facilitate direct use during the operation (Fig. 5d). (4) Plates and screw preparation: The orginal plates, pre-reshaped plates and all screws were prepared in two sets that were sterilized for direct use during the operation. Meanwhile, the 3D-printed model of the original tibial plateau fracture and the model after reduction and fixation using plates and screws were also brought into the operating room for reference during the operations. The 3D printing procedure and preoperative simulation were completed in our 3D printing Lab during anticoagulant and venous thrombolytic therapy of the lower extremity DVT (24.65 ± 4.39 days).

### Surgical technique

Operations were performed under spinal or general anesthesia, a tourniquet on the proximal thigh was required. The supine position and antero-middle approach were chosen for tibial plateau fractures with relatively complete medial and lateral columns (type V). For comminuted fractures of the medial column with knee joint dislocation (type IV) and three-column fractures extending to the metaphysis (type VI), the floating position and posteromedial inverted “L” approach combined with the antero-middle approach were chosen. Using the posteromedial inverted “L” approach, the gastrocnemius medialis, tibial nerve and popliteal blood vessels were laterally pulled and protected using a hook, then the soleus and popliteal muscles were retracted and bluntly stripped with a periosteal elevator to expose the fracture fragments of the posterior column. The antero-middle approach was used to expose the fracture fragments of the medial and lateral columns from both sides of the patellar ligament, and to expose the knee joint.

For the conventional planning group, the intraoperative identification and reduction of the fragments mainly based on preoperative 3D CT images and the clinical experience of the orthopaedic surgeons. The fracture fragments were reduced and temporarily fixed with K-wires. Then for type V fracture, two medial and lateral proximal tibial locking plates were used for final fixation. Sometimes, the medial plate needs to be intraoperatively reshaped to match the surface of the reduced tibial fracture fragments. For type IV fracture, one proximal tibial medial locking plate and one posteromedial locking plate or straight reconstruction locking plate were used for fixation, two plates need always to be intraoperatively reshaped. For type VI fractures, two medial and lateral proximal tibial locking plates and one posterior straight reconstruction locking plate or distal radius “T” type locking plate were used for fixation, the medial and posterior plates often need to be intraoperatively reshaped. If the intraoperative implantation of the plates did not match the surface of the reduced tibial fracture fragment, the plates need to be reshaped several times, but rarely more than five times. Subsequently, the length of the screw track was measured and the corresponding screws were implanted.

For the planning with 3D printing group, the fragments were carefully confirmed and separated according to the real-time reference of the original 3D printing fracture model. Referring to the preoperative fracture model after reduction and fixation, the main fracture fragments were firstly reduced and temporarily fixed with K-wires according to the preoperative design, after which the other fragments were reduced and fixed. The pre-reshaped locking plate such as the “T” plate or straight reconstruction plate and the anatomical locking plates were implanted. The intraoperative implantation of the preoperative pre-reshaped plates needed to match the surface of the reduced tibial fracture fragments; if not, it was necessary to check and adjust the fracture reduction again until the plates attached to the reduced fracture. According to the preoperative planning, the corresponding screws were directly selected for fixation without the intraoperative length measurement.

If necessary, for both groups, allogeneic and autogenous bone-grafts were implanted into the bone defects. After satisfactory fracture reduction and fixation under “C” arm X-ray fluoroscopy, the incisions were closed. All the operations in this study completed by the same orthopedic team with one senior orthopedic doctor (Dr. Honglue Tan).

**Figure 6:**
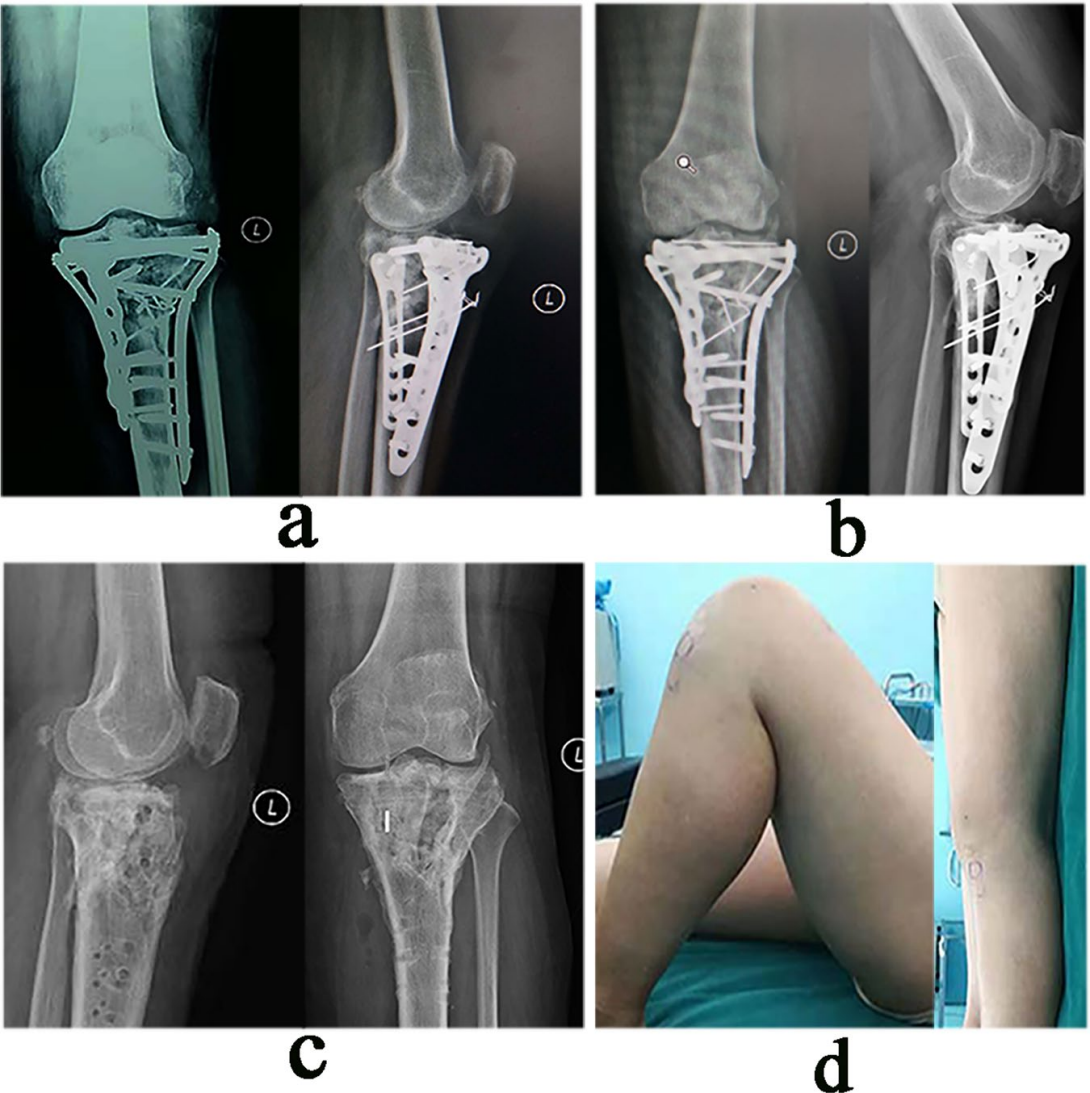
The radiography and knee function after operation. (**a**) The postoperative X-ray showing satisfactory fracture reduction and internal fixation which were consistent with the pre-operative simulation. (**b**) X-ray at 14 months postoperatively showing bone-union with no displacement of implants or collapse of the joint surface. (**c**) X-ray at 6 months postoperatively after plate removal showing normal shape and structure of the tibial plateau. (**d**) The patient had satisfactory knee function with an HSS score of 85.

The post-operative rehabilitation were performed after recovery from anesthesia, the patients were encouraged to perform isometric quadriceps exercises. Passive and active flexion and extension of the knee joint were started as early as possible; the flexion angle was gradually increased with time. Using a crutch, patients were allowed ambulation without weight-bearing 2 weeks post-operation, after which partial and complete weight-bearing was assessed according to the level of fracture healing. Patients were followed up at 1, 3 and 6 months and at 1 year. Thereafter, they were followed up annually (Fig. 6).

### Outcome measures

The assessment parameters included surgical duration, blood loss and the number of fluoroscopy under “C” arm X-ray machin during operation. Surgical duration was defined as the time from skin incision to closure. Total intraoperative blood loss was the collected blood from the suction bottle and blood from the weighed gauze^8^. Complications including infection, implant failure, traumatic arthritis (posttraumatic arthritis was described as painful range of motion with radiographic evidence of a narrowed joint space^9^), poor incision healing, deep venous thrombosis (DVT) and bone nonunion were also recorded. At the end of follow-up, the quality of fracture reduction was evaluated according to Rasmussen’s radiological score, which was graded as excellent (18 points), good (12 points), fair (6 points) or poor (0 points)^10^. Knee function was evaluated using the hospital for special surgery (HSS) scoring system^11^. For HSS scores, a score of ≥ 85 points is excellent, 70–84 points is good, 60–69 points is fair, and ≤59 points is poor^11^. All patients were examined in the outpatient department following their surgery. The radiological assessments were performed by a radiologist who did not participate in the treatment.

### Statistical analysis

Results were analyzed using SPSS statistical software 15.0 (SPSS Inc., Chicago, IL, USA). Qualitative variables are summarized as numbers and percentages, and quantitative variables are summarized as mean ± SD. The normality of distribution for continuous numeric variables was assessed by Kolmogorov-Smirnov test. The chi-square test was used for qualitative data. Independent *t* test or the Mann-Whitney test was used for quantitative data according to normally distributed or not. A *P* < 0.05 was considered to be statistically significant.

## Results

### Clinical demographic data

In the conventional planning group, there were 22 patients (13 males and nine females) with mean age of 43.00 ± 7.86 years (ranged from 29 to 55 years); BMI was 25.02 ± 1.72 kg/m^2^ (ranged from 21.97 to 27.85 kg/m^2^); 12 had fractures in the right knee and ten had fractures in the left knee. Falling from height was the cause in nine cases, traffic accidents in eight cases and other causes in five cases. The time from admission to hospital to surgery was 26.91 ± 5.65 days (ranged from 21 to 37 days). According to the Schatzker classification, nine patients were type IV, six were type V, and seven were type VI. The mean follow-up time was 23.45 ± 8.28 months (ranged from 12 to 36 months). In the planning with 3D printing group, there were 20 patients (14 males and six females) with mean age of 42.85 ± 7.71 years (ranged from 29 to 55 years); BMI was 24.96 ± 2.03 kg/m^2^ (ranged from 20.76 to 28.04 kg/m^2^); 12 had fractures in the right knee and eight had fractures in the left knee. Falling from height was the cause in eight cases, traffic accidents in seven cases and other causes in five cases. The time from admission to hospital to surgery was 24.65 ± 4.39 days (ranged from 21 to 35 days). Based on the Schaktzer classification, six were type IV, eight were type V and six were type VI. The mean follow-up time was 25.40 ± 6.87 months (ranged from 18 to 36 months). Additionally, there were no significant between-group differences in the number of plates used and the choice of surgical approach. The demographic data are summarized in Table 1 and were comparable (*P* > 0.05).

### Peri-operative clinical parameters

As shown in Table 2, in planning with 3D printing group, the preoperative software time was 48.50 ± 4.79 min (ranged from 40 to 60 min), the printing time for the 3D model was 516.50 ± 84.18 min (ranged from 400 to 680 min), and the preoperative simulation time (including plate precontouring) was 118.50 ± 15.31 min (ranged from 100 to 150 min). The mean intraoperative time in the planning with 3D printing group was 127.25 ± 8.03 min (ranged from 120 to 140 min), which was significantly lower than that of the conventional planning group (152.50 ± 29.63 min [ranged from 100 to 200 min]; *P* = 0.001). The intraoperative mean blood loss in the planning with 3D printing group was significantly less than that in the conventional planning group (202.50 ± 36.26 ml [ranged from 150 to 280 ml] versus 266.36 ± 49.04 ml [ranged from 180 to 360 ml] respectively; *P* = 0.001). Finally, the number of intraoperative fluoroscopy was performed significantly less in the planning with 3D printing group (6.55 ± 1.09 [ranged from 5 to 9]) than in the conventional planning group (11.00 ± 1.83 [ranged from 9 to 14]; *P* = 0.001). Number of intraoperative plate reshaping in the conventional planning group was 2.91 ± 1.06 (ranged from 1 to 5).

**Table 2:** Clinical and functional data between two groups.

		Conventional planning group (n = 22)	Planning with 3D printing group (n = 20)	*P*-value
Software time (min)		—	48.50 ± 4.79	n/a
3D printing time (min)		—	516.50 ± 84.18	n/a
Pre-operative simulation (including plate precontouring) (min)		—	118.50 ± 15.31	n/a
Operation time (min), M ± SD		152.50 ± 29.63	127.25 ± 8.03	0.001^b^
Blood loss (ml), M ± SD		266.36 ± 49.04	202.50 ± 36.26	0.001^b^
No. of fluoroscopy (n), M ± SD		11.00 ± 1.83	6.55 ± 1.09	0.001^b^
No. of plate reshaping (n), M ± SD		2.91 ± 1.06	0	n/a
Rasmussen’s score, n (%)				
	Excellent	10 (45.45)	15 (75.00)	0.05^a*^
	Good	12 (54.55)	5 (25.00)	
	Fair	0 (00.00)	0 (00.00)	
	Poor	0 (00.00)	0 (00.00)	
Complication, n (%)		7 (31.82)	3 (15.00)	
HSS score, M ± SD		79.09 ± 6.75	86.05 ± 7.67	0.003^b^
HSS score, n (%)				
	Excellent	10 (45.45)	14 (70.00)	0.083^a*^
	Good	10 (45.45)	4 (20.00)	
	Fair	2 (09.10)	2 (10.00)	
	Poor	0 (00.00)	0 (00.00)	

^a^chi-squared test, ^b^Mann-Whitney test, *the excellent rate between groups. M ± SD: mean ± standard deviation; No.: number; n: patient number.

### Post-operative parameters

At 6-month follow-up, X-ray films showed that all patients in two groups had bone union. As shown in Table 2, at the end time of follow-up, the proportion of patients who scored excellent in the Rasmussen’s radiological score for fracture reduction in the planning with 3D printing group was significantly higher than that in the conventional planning group (75% and 45.45% respectively; *P* = 0.05). The rate of complication in the planning with 3D printing group and the conventional planning group was 15% (3/20) versus 31.82% (7/22), respectively. In the planning with 3D printing group, two patients developed traumatic arthritis and one had superficial infection; while in the conventional planning group, four patients had traumatic arthritis, one had superficial infection and two had incision necrosis. No other complications such as bone nonunion, neurovascular injury, fixation failures, and DVT were found in either group.

### Post-operative functional assessment

As shown in Table 2, the mean HSS score for the planning with 3D printing group was significantly higher than that of the conventional planning group (86.05 ± 7.67 and 79.09 ± 6.75, respectively; *P* = 0.003). However, there was no significant difference in the rate of patients who scored excellent in the HSS score between the groups (70.00% in the planning with 3D printing group and 45.45% in the conventional planning group; *P* = 0.083).

## Discussion

3D-printing technology, based on digital modeling to construct objects by layer-by-layer printing using adhesive materials, has been rapidly developed in the medical field and applied in orthopedics^6,12,13^. 3D-printing technology can produce an individualized 1:1 solid prototype of the fracture, based on which enabling surgeons to observe the structural morphology of the fracture fragments. In turn, surgeons can use the model in preoperative planning to more accurately determine the surgical approach needed to successfully reduce and fix the fracture fragments. According to literature reports, this technology is beneficial in improving the accuracy of the orthopedic operation, reducing blood loss, shortening operation times and in promoting postoperative functional recovery. Yang *et al*. applied a 3D print-assisted design to the surgical treatment of a tri-malleolus fracture, which accurately displayed the morphology of the fracture and contributed to preoperative planning. Results also indicated that the 3D print-assisted design significantly reduced intraoperative blood loss and shortened the operation time; Meanwhile, the 3D printing model also was beneficial to the communication between doctors and patients regarding the surgical planning^14^. Hung *et al*. used 3D printing technology to generate preoperative models of pelvic fractures and showed that compared with conventional preoperative planning using X-ray and CT images, 3D print-assisted preoperative planning was more accurate in identifying the exact type of fracture^15^. Furthermore, for fresh tibial plateau fractures, the application of 3D printing technology has also been reported. Lou *et al*. used 3D printing to generate tibial plateau fracture models, and carried out “pre-surgery” on the models to determine the shape of the fixed plates and the direction and length of the fixed screws. Results showed that compared with the conventional preoperative planning guided by CT images, preoperative use of 3D printing models decreased the operation time, the intraoperative blood loss and reduced the fluoroscopy time. Furthermore, postoperative CT also confirmed that the fracture fragments were reduced and the position of the implants was appropriate^3^. Silvio *et al*. prospectively evaluated the clinical follow-up efficacy of using 3D printing techniques to assist minimally invasive reduction and fixation of tibial plateau fractures, and showed that no significant differences in functional recovery existed between patients that had preoperative 2D scans and those that utilized 3D printing technology. Importantly, the operation time and the radiographic exposure time of patients that utilized 3D printing preoperatively were reduced^4^.

The current study mainly explored the auxiliary role of 3D printing in the preoperative planning and intraoperative surgical process of old and complex plateau fractures. The results showed that an individualized solid model of the fracture with an equal ratio to the actual fracture produced by 3D printing techniques can help surgeons accurately observe and analyze the morphology and displacement of fragments. Meanwhile, through preoperative surgical simulation on the 3D printing model, the reduction steps of the fragments, placement and pre-reshaping of the plates, and the measurement of the screw length can be accurately determined and recorded. During the operation, the anatomical reduction and the fixation of the fragments can be performed according to the preoperative planning design, thus in our study, the operation time and intraoperative blood loss in the planning with 3D printing group were less than that in the conventional planning group. Postoperative follow-up results also showed that the articular surface and plateau height remained viable, and the excellent rate of function was higher than that in the conventional planning group. Taken together, the results suggest that 3D-printed fracture models facilitate the treatment of old and complex tibial plateau fractures via a more accurate and personalized approach, thus confirming its effectiveness for this type of fracture.

Preoperative simulation using 3D printing models has the following advantages: (1) The model, with an equal ratio of the original fracture, allows surgeons to clearly understand the morphology of the fracture fragments, the fracture collapse and any hidden fragments that might be present, all which help to determine an accurate judgment of the fracture type, the correct surgical approach and the choice of intraoperative body position. (2) The effectiveness of plate fixation largely depends on the shape matching between the plates and reduced fractures. However, for complex tibial plateau fractures, a common problem is that the plates do not always attach to the bone surface, and therefore requires repeated reshaping and position adjustment of the plate during the operation. We propose that if a 3D-printed model is used to simulate the operation before surgery, not only can the appropriate plate be selected and reshaped in advance, but the proper fixation position and the length and direction of the screw can also be determined preoperatively. Pre-shaping plates and screws should be prepared in advance and disinfected for direct use during the operation, which decreased the operation time, improved the surgical efficiency and reduced the radiation exposure. (3) The 3D-printing model, before and after reduction and fixation, can be brought into the operating room and used in real-time during the operation to guide the surgeon and to improve the operation efficiency. (4) Through 3D-printing, doctors can explain the treatment planning to patients, helping patients to understand and cooperate with doctors, and thus making the doctor-patient relationship more harmonious.

However, problematic several disadvantages still exist for this technique: (1) The price of 3D-printing planning with 3D printing is relatively high, which increases the hospitalization cost. (2) The printing of the model is time consuming and therefore the technology is only suitable for old and complex fractures. (3) The printed model can only display bone shape and does not consider soft tissue, such as blood vessels, ligaments and cartilage; therefore, the preoperative planning should be designed in combination with MRI and vascular imaging findings.

The following items should be noted for this technique: The printed model must be prepared at a ratio of 1:1 with the original tibial plateau fracture. During simulated operation on the model, the separation and reduction of the fragments should be done gently and carefully, thus avoiding re-splitting of individual fragments or splitting of non-fractured areas of the model. During the actual operation, preoperative simulation procedure and MRI findings are used to determine intraoperative body position and skin incision location, to identify the reduction sequence of fragments. The original fracture model before fragment reduction and the model after reduction and fixation should be used as a real-time intraoperative reference. Finally, 3D-printing technology can only be used to assist surgery, perioperative management cannot be ignored such as anticoagulation, skin incision management and postoperative functional rehabilitation.

There are some limitations in our study, which may have a slight impact on the conclusion. First, the study is a retrospective and non-randomized-controlled design, and the sample size enrolled is relatively small, which may limit the reliability of the results. Second, in this study, the patients in the conventional planning group and in the planning with 3D printing group were operated at different time intervals, which may lead to a bias of the clinical results due to surgeons’ learning curve of the 3D printing techniques. Third, this study mainly focus on the peri-operative results and short-term clinical outcomes, which may introduce a bias effect in the observed complications. Finally, old Schatzker IV, V and VI tibial plateau fractures in this study were put together to explore the clinical efficacy of pre-operative simulation using a 3D printing model for operation, which did not take into account the different surgical procedures for each type of fracture. It would be more reasonable to perform different clinical studies for each type. However, in our case, the small sample size limited the application of this study design. So the heterogeneity of fracture types may also have a bias effect on the results. Therefore, according to aforementioned limitations, the high-quality prospective randomized-controlled studies with a larger patient population from multicentre and long-term follow-up should be performed to further assess the clinical application and effectiveness of 3D printing technique in the management of old and complex tibial plateau fractures.

In conclusion, 3D-printing models and surgical simulation provided surgeons with a tangible pre- and intra-operative evaluation of old and complex tibial plateau fractures. This technology may be an effective method for the treatment of this type of fracture, and result in a shorter operation time, less intraoperative blood loss and fluoroscopy times, and a higher quality of fracture reduction. However, the conclusion of this study still needs to be further assessed by a prospective randomized-controlled study.

## Data Availability

The datasets generated and analysed during the current study are available from the corresponding author on reasonable request.
